# Modified Lichong decoction intervenes in colorectal cancer by modulating the intestinal flora and the Wnt/β-catenin signaling pathway

**DOI:** 10.1007/s00432-024-05763-w

**Published:** 2024-05-06

**Authors:** Longhui Liu, Mengmeng Zhao, Xiaomeng Lang, Sujie Jia, Xin Kang, Yue Liu, Jianping Liu

**Affiliations:** 1 Graduate School of Hebei University of Chinese Medicine, Shijiazhuang, 050091 Hebei China; 2 Department of Spleen and Stomach Diseases, Hebei Provincial Hospital of Traditional Chinese Medicine, Shijiazhuang, 050013 Hebei China

**Keywords:** Colorectal cancer, Lichong decoction, Intestinal flora, Wnt/β-catenin, Cell cycle, Epithelial–mesenchymal transition

## Abstract

**Background:**

The pathogenesis and treatment of colorectal cancer (CRC) continue to be areas of ongoing research, especially the benefits of traditional Chinese medicine (TCM) in slowing the progression of CRC. This study was conducted to investigate the effectiveness and mechanism of action of modified Lichong decoction (MLCD) in inhibiting CRC progression.

**Methods:**

We established CRC animal models using azoxymethane/dextran sodium sulfate (AOM/DSS) and administered high, medium, or low doses of MLCD or mesalazine (MS) for 9 weeks to observe MLCD alleviation of CRC. The optimal MLCD dose group was then subjected to metagenomic and RNA sequencing (RNA-seq) to explore the differentially abundant flora and genes in the control, model and MLCD groups. Finally, the mechanism of action was verified using WB, qRT‒PCR, immunohistochemistry and TUNEL staining.

**Results:**

MLCD inhibited the progression of CRC, and the optimal effect was observed at high doses. MLCD regulated the structure and function of the intestinal flora by decreasing the abundance of harmful bacteria and increasing that of beneficial bacteria. The differentially expressed genes were mainly associated with the Wnt/β-catenin pathway and the cell cycle. Molecular biology analysis indicated that MLCD suppressed the Wnt/β-catenin pathway and the epithelial–mesenchymal transition (EMT), inhibited abnormal cell proliferation and promoted intestinal epithelial cell apoptosis.

**Conclusion:**

MLCD mitigated the abnormal growth of intestinal epithelial cells and promoted apoptosis, thereby inhibiting the progression of CRC. This inhibition was accomplished by modifying the intestinal microbiota and disrupting the Wnt/β-catenin pathway and the EMT. Therefore, MLCD could serve as a potential component of TCM prescriptions for CRC treatment.

## Introduction

Colorectal cancer (CRC) is a malignancy of the colon or rectum that is characterized by abnormal cell proliferation that projects into the intestinal cavity. CRC ranks second in incidence and third in cancer-related deaths, and accounts for approximately one-tenth of all patients with cancer and fatalities (Sung et al. 2021). Notably, CRC is insidious in onset and prone to rapid progression in advanced stages and severe metastasis. In fact, distant invasion and metastasis account for up to 90% of deaths from CRC (Liu et al. 2017). Addressing the malignant progression and aggressiveness of CRC presents urgent and difficult challenges in a clinical context. Recently, TCM has gained prominence due to its role in hindering CRC progression. Thus, investigating herbal formulas that inhibit CRC progression is crucial for alleviating patient suffering.

Pathological factors such as flora disorders, genetic mutations, and inflammation contribute to CRC development (Grivennikov et al. 2012). Flora disturbances, particularly changes in the structure and function of flora, are instrumental in promoting tumor growth (Garrett 2019). Dysbiosis induces CRC by stimulating inflammation, damaging DNA, promoting tumor cell growth, and activating various signaling pathways (Fong et al. 2020). The distribution of intestinal microbial communities can be monitored through metagenomic analysis, and the function of bacterial groups can be assessed to understand how changes in flora affect CRC. Studies have suggested that the intestinal flora can regulate the transcriptome and influence CRC development (Pan et al. 2018). Disease-associated genes and gene regulatory networks can be explored at the transcription level using RNA-seq. Thus, the combined use of metagenomics and RNA-seq is an effective approach for understanding disease development and assessing the effectiveness of treatments.

The Wnt pathway, which is prevalent in both invertebrates and vertebrates, is a highly conserved evolutionary signaling pathway. Wnt/β-catenin, which is part of the canonical Wnt signaling pathway, can lead to the development and invasive metastasis of various tumors, including CRC, when abnormally activated (Krishnamurthy and Kurzrock 2018). Prior research indicates that approximately 90% of CRC cases involve Wnt/β-catenin mutations, and over 80% exhibit abnormal β-catenin accumulation in the nucleus (Sebio et al. 2014). The EMT, a process by which epithelial cells are converted into mesenchymal cells (Brabletz et al. 2018), is vital for CRC development, invasion, and metastasis. During the EMT, cells shift from a stationary, adherent state to a mobile, metastatic state (Yeung and Yang 2017). An essential feature of the EMT is the loss of epithelial cells, as shown by E-cadherin expression, accompanied by an increase in mesenchymal cells, as indicated by increases in N-cadherin and vimentin expression (Jin and Wu 2019). Given the important role of the EMT and the Wnt/β-catenin pathway in CRC progression, their inhibition could be crucial in inhibiting CRC.

TCM is an integral part of China’s exceptional ancestral culture, boasting a history of more than 3000 years. The unique formulas, which are composed of various medicinal herbs, are often employed for numerous clinical ailments. Contemporary studies increasingly highlight the potential of these formulas for the effective prevention and treatment of intestinal tumors. The exploration of the preventive and therapeutic qualities of some classical Chinese herbal formulas is still in progress. MLCD, a derivative of a classic TCM recipe, is traced to the text ‘Yi Xue Zhong Zhong Can Xi Lu’ by the Qing-era doctor Zhang Xichun. The core Lichong decoction is known to enhance qi, stimulate blood circulation, regulate menstruation, and alleviate blood stagnation. The formula is predominantly used for abdominal gynecological obstructions. The constituents of MLCD are sheng huang qi, dang shen, bai zhu, sheng shan yao, huang lian, huang bai, san leng, e zhu, ji nei jin, and bai hua she she cao. MLCD is a flexible deviation from the original formula based on the causes and pathogenesis of CRC.

The aim of this study was to investigate the effectiveness of MLCD in inhibiting CRC and to explore the underlying mechanism. The use of metagenomic sequencing and RNA-seq allowed us to identify bacteria and monitor gene expression variations across groups. The identification of related genes and proteins was achieved through molecular biology techniques.

## Materials and methods

### Chemical reagents

Azoxymethane (Sigma, United States), dextran sulfate sodium (36–50 kDa, MP Biomedicals, California, United States), TriQuick Reagent (Solarbio, Beijing, China), Ethanol Absolute (Tianjin, China), an RNA 6000 Nano LabChip Kit (Agilent, CA, USA, 5067–1511), and antibodies against E-cadherin (#20874–1-AP, Proteintech), N-cadherin (#22018–1-AP, Proteintech), vimentin (#10366–1-AP, Proteintech), C-myc (#18583s, Cell Signaling Technology, United States), β-catenin (#8480s, Cell Signaling Technology, United States), CyclinD1 (#55506s, Cell Signaling Technology, United States), and Anti-GAPDH (#AP0063, Proteintech) were used. TNF-α, IL-1β, L-10, and IL-6 ELISA kits were obtained from Senxiong (Shanghai, China).

### Animals and feeding environment

We acquired 60 male C57BL/6 J mice, aged 6–8 weeks and weighing 18–22 g, from SPF (Beijing) Biotechnology Co., Ltd. (License No. SCXK (Jing) 2019–0010). The mice were housed at the Laboratory Animal Centre of Hebei University of Chinese Medicine under controlled conditions (22 ± 2 °C, 55 ± 5% humidity, a 12-h light/dark cycle, and a standard diet). We recorded the body weights of mice weekly. During this study, we strictly adhered to the animal welfare standards and procedures outlined by the National Institutes of Health’s Care and Use of Laboratory Animals. The Animal Ethics Committee of Hebei University of Traditional Chinese Medicine reviewed and approved our study (Authorization No. DWLL202212013).

### Preparations of MLCD

MLCD is a mixture of ten types of TCM granules. The granules are sheng huang qi (9 g), dang shen (6 g), bai zhu (6 g), sheng shan yao (15 g), huang lian (12 g), huang bai (12 g), san leng (9 g), e zhu (9 g), ji nei jin (9 g), and bai hua she she cao (20 g). These granules were obtained from Guangdong Yifang Pharmaceutical Co., Ltd., in Guangdong, China. The granules were finely crushed and dissolved in warm water for consumption.

### Establishment of the CRC model and treatment

**Fig. 1:**
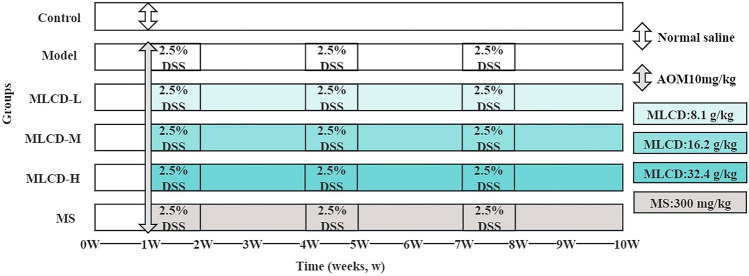
Overview diagram of the experimental process

After a 7-day dietary adaptation period, 60 mice were randomly assigned to six groups, with ten mice in each group: control, model, MLCD-L, MLCD-M, MLCD-H, and MS. Control mice received an intraperitoneal saline injection on the first day of the second week, and they were provided with regular drinking water for the entire experiment. The remaining 50 mice were given intraperitoneal injections of 10 mg/kg AOM on day one of week two; furthermore, their drinking water was supplemented with 2.5% DSS for seven consecutive days in weeks 2, 5, and 8, alternating with saline during weeks 3, 4, 6, 7, 9, and 10. Starting on day one of week two, the control group was given a saline ‘gavage’. The doses for the other groups were as follows: MLCD-L (8.1 g/kg), MLCD-M (16.2 g/kg), MLCD-H (32.4 g/kg), and MS (300 mg/kg) (Fig. 1).

Following the 9-week intervention described above, the mice were euthanized via carbon dioxide asphyxiation (Fig. 1). Dissection was carried out, and a cecum-anal sample was removed. This removal was followed by opening the mouse longitudinally and photographic documentation, as well as measurements of tumor counts and bowel length. Specific intestinal tumor tissue sections were isolated and preserved in 4% paraformaldehyde (PFA). Finally, the feces (three to four samples) and intestinal tumor tissues were transferred to 1.5 ml EP tubes, flash-frozen in liquid nitrogen, and subsequently stored at -80 °C.

### Hematoxylin eosin (HE) staining assay

The intestinal tissues were fixed with 4% paraformaldehyde, removed and dehydrated using an automated dehydrator. We embedded these tissues in paraffin to create wax blocks. We then cut the wax blocks into approximately 4 μm thick slices using a paraffin slicer and stained them with HE. Finally, we observed any pathological changes in the intestinal tissue under a microscope.

### Metagenomic sequencing

Three mouse fecal samples from the control, model, and optimal MLCD dose groups were chosen randomly for metagenomic sequencing, respectively, based on the pharmacodynamic results. Total RNA was extracted from the samples using the Fecal Genome DNA Extraction Kit (AU46111-96, BioTeke, China). The DNA concentration was measured, DNA fragmentation that met the concentration requirements was detected, and the fragmentation product size was determined. DNA library construction for compliant fragment products (typically 200–500 bp) was carried out using the TruSeq Nano DNA Library Preparation Kit-Set (#FC-121–4001, Illumina, USA) according to the manufacturer’s instructions. Subsequently, the metagenomic libraries were sequenced using PE150 on an Illumina NovaSeq 6000 platform. Differentially abundant species were identified using the Wilcoxon test, with *P* < 0.05 and |log2-fold change|> 1 indicating significance. Kyoto Encyclopedia of Genes and Genomes (KEGG) analysis was used to determine microbiological functions.

### RNA-seq

We selected intestinal tumor tissue from nine mice (as described in Sect. “Metagenomic sequencing”) and sent the tissue to Biotree (Shanghai Biotree Co., Ltd., Shanghai, China) for RNA-seq. We assessed the total RNA quantity, purity, and integrity for RNA quantification and qualification. Using this technology, we constructed a cDNA library from the pooled RNA of intestinal tumor tissue samples from C57BL/6 mice. We then sequenced this library using the Illumina NovaSeq™ 6000 sequencing platform. To ensure high-quality sequences, we filtered the reads using Cutadapt and assessed them with FastQC. Using the HISAT2 package, we aligned these reads to the mouse reference genome. After finalizing the transcriptome, we calculated the FPKM value to determine the amount of mRNA expression. We estimated the expression levels of all transcripts using StringTie and Ballgown. We employed DESeq2 software to analyze the differentially expressed genes in two distinct groups. Any genes satisfying the criteria of having a fold change ≥ 2 and a *P* value < 0.01 were considered differentially expressed.

### Western blot (WB) analysis

We removed intestinal tissues from the control, model, and MLCD groups from a – 80 ℃ environment and centrifuged them post-RIPA lysis digestion. Using the BSA method, we measured the concentration of proteins and then separated equal amounts of protein via 10% SDS‒PAGE (Boster, Wuhan, China). Afterward, the proteins were transferred to a PVDF membrane (Millipore, USA). After incubation for 90 min at room temperature, the membrane was blocked with 5% skim milk. We then incubated the membranes with primary antibodies overnight at 4 °C. The membrane was incubated for 1 h with secondary antibodies, followed by washing with TBST three times and TBS once. After the addition of the ECL luminescent reagent (Vazyme, Nanjing, China), we analyzed the protein bands. Image Lab was utilized for grayscale value analysis of the protein bands.

### Quantitative real-time PCR (qRT-PCR) analysis

We extracted total RNA from intestinal tissues using TriQuick Reagent according to the manufacturer’s instructions (Solarbio, Beijing, China). Next, we synthesized cDNA using a reverse transcription kit (GeneCopoeia, Guangzhou, China). We then conducted qRT‒PCR amplification using iQ5 Real-Time PCR (Applied Biosystems, USA) with 2 × SYBR Green qPCR Master Mix (No ROX) (Servicebio, Wuhan, China). Finally, we calculated the relative mRNA expression of the genes using the 2-ΔΔCT method.

### Immunohistochemistry assay

Paraffin sections (described in Sect. “Hematoxylin eosin (HE) staining assay”) were prepared, deparaffinized, hydrated, and subjected to antigen repair and sealing. The sections were then coated with PCNA and Ki67 antibodies and incubated overnight at 4 °C. After washing three times with PBS, secondary antibodies were applied at a 1:50 dilution, and the sections were incubated at room temperature for 20 min. Afterward, the sections were washed again with PBS and developed with DAB for visualization. Hematoxylin was then used for re-staining and sealing. Finally, the sections were evaluated and photographed using a microscope.

### TUNEL assay

We detected apoptosis in intestinal tumor tissue using the TUNEL assay according to the manufacturer’s instructions. We prepared Paraffin sections (described in Sect. “Hematoxylin eosin (HE) staining assay”) and incubated them with TUNEL solution for one hour at 37 °C in the dark. Afterward, the tissues were washed three times with PBS. We then re-stained the cell nuclei by adding DAPI solution dropwise and incubating the cells for 10 min at room temperature in the dark. We washed the sections once more, dehydrated and sealed the sections, and then examined and photographed them using a fluorescence microscope. For statistical analysis, we used Image J software.

### Determination of TNF-α, IL-1β, IL-6, and IL-10 levels in intestinal tissue

Kits were used to measure the expression levels of TNF-α, IL-1β, IL-6, and IL-10 in intestinal tissue according to the manufacturer’s instructions.

### Statistical analysis

We analyzed the experimental data using GraphPad Prism 8.0 and SPSS 21. The results are expressed as the mean plus or minus the standard deviation. We used one-way analysis of variance (ANOVA) to compare the multiple groups. A *P* value less than 0.05 indicated a statistically significant difference.

## Results

### Efficacy of MLCD inhibition of CRC progression

**Fig. 2:**
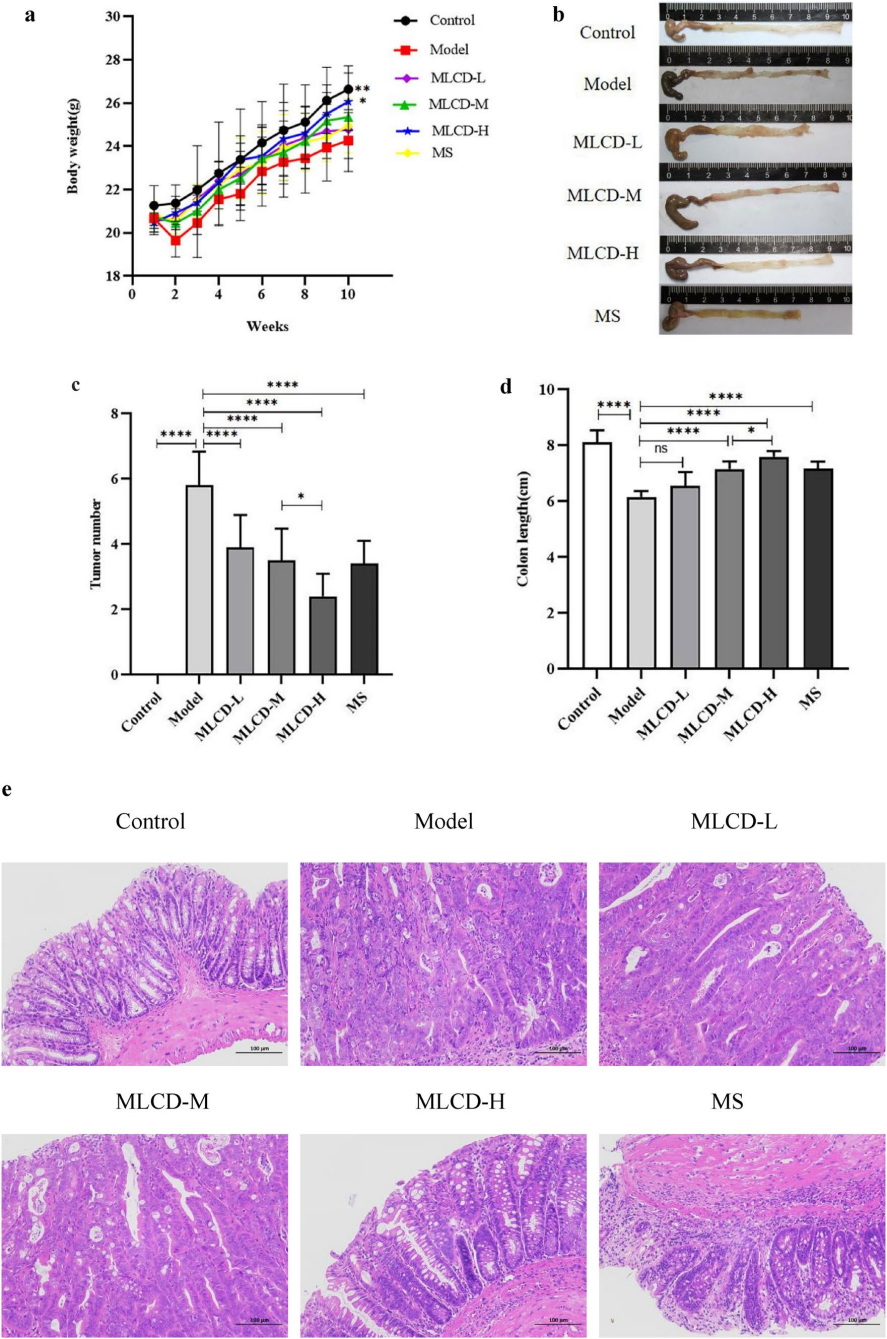
Efficacy of MLCD inhibition of CRC. **a** Weekly body weight changes in the mice. **b** Typical images of intestinal tumors from the six groups. **c** Number of tumors. **d** Length of the colon. **e** H&E staining of intestinal tumor tissue sections. (*P < 0.05, ****P < 0.0001, the difference is statistically significant)

Before the experiment began, there was no significant difference in weight among mice in the six groups. As the experiment continued, there was an increase in weight in the control group. Moreover, compared with the control group, the model group, which consumed 2.5% DSS for weeks 2, 5, and 8, exhibited either no change in weight or weight loss. This group also exhibited symptoms such as bloody stools and diarrhea. Despite consuming 2.5% DSS, the MLCD-L, MLCD-M, MLCD-H, and MS groups experienced less weight loss than did the model group, with fewer cases of bloody and loose stools (Fig. 2a). After the experiments ended and the intestines of the mice were dissected, these groups had significantly fewer tumors (Fig. 2b, c) and less intestinal length shortening (Fig. 2d) than the model group. The MLCD-H group showed the greatest improvement. Furthermore, the intestinal tissue of the model group showed substantial heterogeneous hyperplasia in the intestinal glandular epithelium. The glandular structure was irregular, and the arrangement was disordered with a reduced number of cup cells. Treatment with MLCD led to occasional cases of adenoepithelial hyperplasia in the intestinal tissue lamina propria and reduced the number of cuprocytes. Other observations included focal ulceration of the intestinal tissue, a few instances of lymphocytic infiltration, and inflammatory cell infiltration into the muscularis propria (Fig. 2e).

### Effects of MLCD on the intestinal flora

**Fig. 3:**
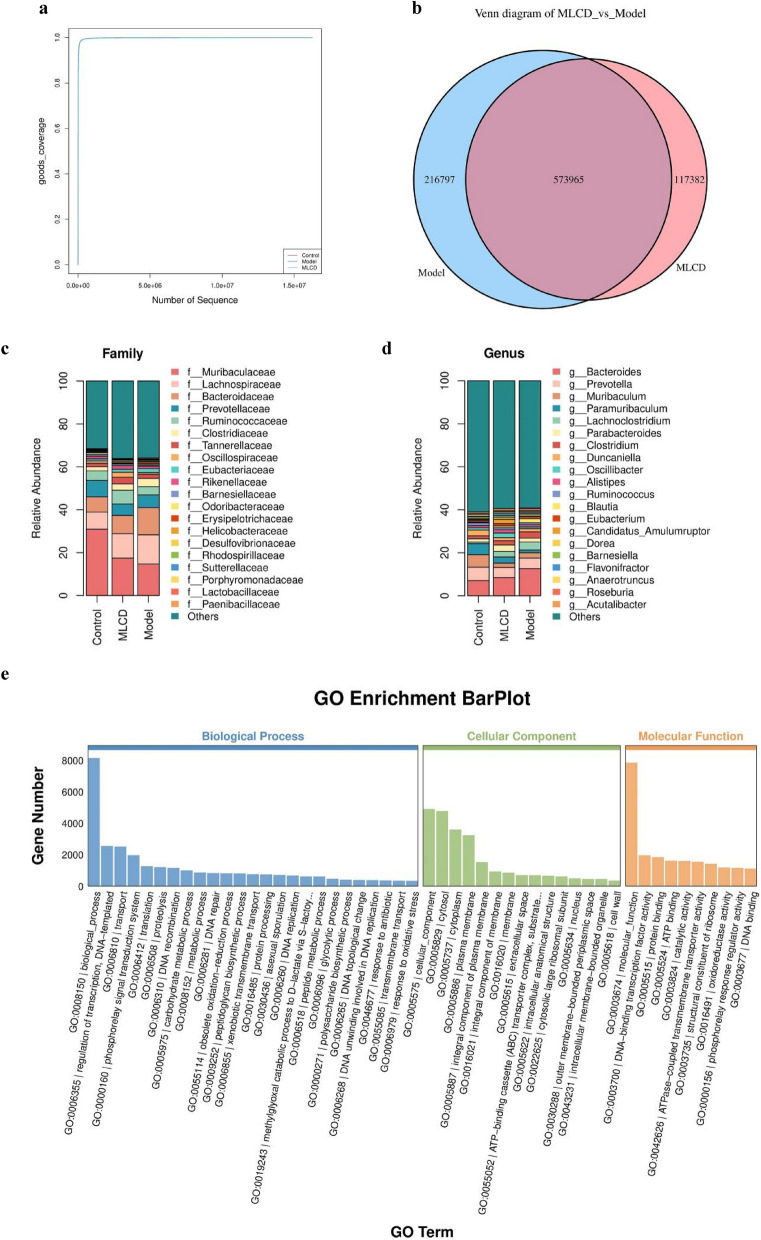

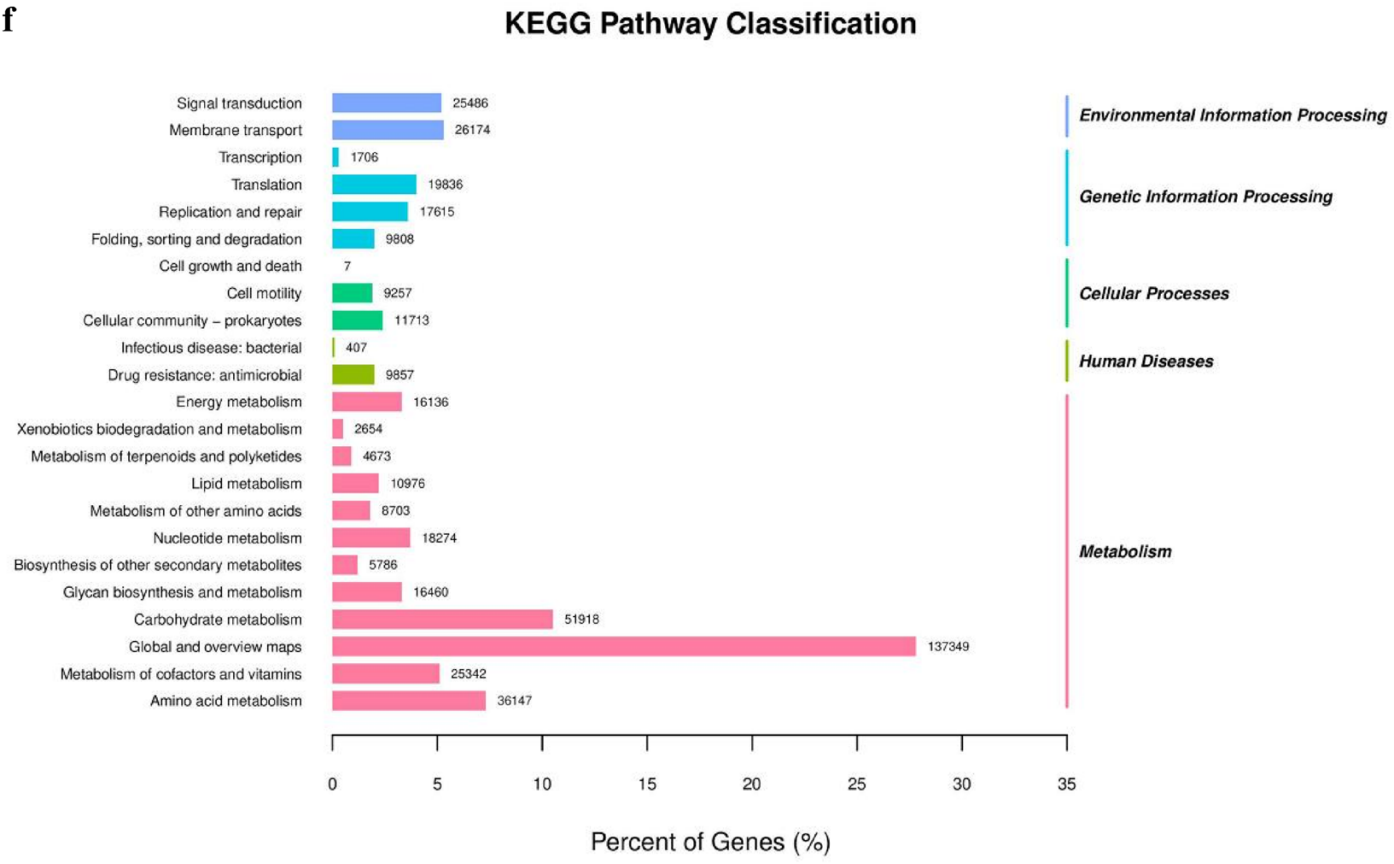
Distribution and functional prediction of the intestinal flora. **a** Goods coverage curves. **b** Venn diagram. **c** Stacked bar plot of species abundance at the family level. **d** Stacked bar plot of species abundance at the genus level. **e** GO enrichment bar plot. **f** KEGG pathway classification

“Goods coverage”, also known as “microbial coverage”, is a measure where a higher value indicates a reduced likelihood of undetected species in a given sample. Our Goods coverage was virtually 1, which suggests that this index faithfully mirrors this sequencing’s findings, thereby representing the sample’s true state (Fig. 3a). The Venn diagram can be used to identify the common and unique unigenes in the model and MLCD groups, which allowed us to visualize the similarity and specificity of the composition of unigenes in the two groups. There were 216,797 unigenes in the model group and 117,382 unigenes in the MLCD group, with a total of 573,965 unigenes in the two groups (Fig. 3b). At the family level, a decrease in Muribaculaceae and an increase in Lachnospiraceae, Bacteroidaceae, and Clostridiaceae were observed in the model group relative to the control group. After MLCD treatment, there was an increased abundance of Muribaculaceae and a decreased abundance of Lachnospiraceae, Bacteroidaceae, and Clostridiaceae (Fig. 3c). At the genus level, compared to the control group, the model group displayed an increase in Bacteroides, Lachnoclostridium, and Clostridium abundance and a decrease in Prevotella and Paramuribaculum abundance. After treatment with MLCD, there was a reduced abundance of Bacteroides, Lachnoclostridium, and Clostridium and an increased abundance of Paramuribaculum (Fig. 3d).

The unigenes had differential expression, which was a significant result of metagenomic sequencing. This result provides a comprehensive view of how these unigenes react differently across various samples or treatments. Subsequently, we conducted GO and KEGG enrichment analyses of the differentially expressed unigenes. GO functional enrichment analysis comprises three facets: biological process (BP), molecular function (MF), and cellular component (CC). BP primarily include elements such as transcription regulation and transport and phosphorelay signal transduction systems. CC mainly pertains to cellular components such as the cytosol and cytoplasm. MF primarily features activities such as DNA-binding transcription factor, protein binding, and ATP binding (Fig. 3e). According to the results of KEGG pathway analysis, environmental information processing includes signal transduction, while genetic information processing includes transcription. Cellular processes were correlated with cell motility (Fig. 3f).

### Effect of MLCD on the intestinal RNA-seq data from CRC mice

**Fig. 4:**
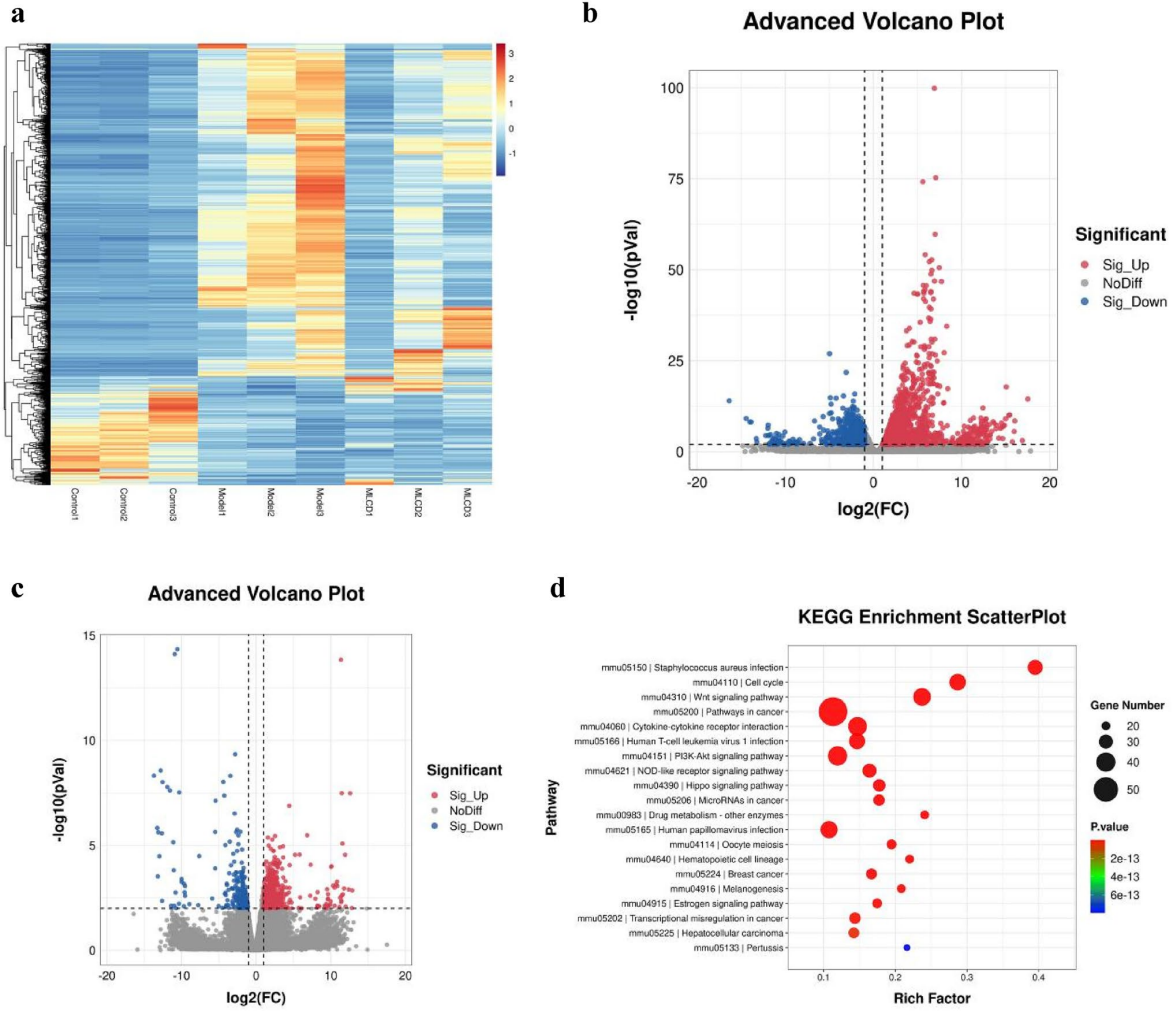
**a** Heatmap analysis of DEGs in the three groups. **b** Volcano plots of DEGs in the model and control groups (n = 3). **c** Volcano plots of DEGs in the MLCD and model groups (n = 3). **d** KEGG analysis of DEGs in the three groups

Differentially expressed genes (DEGs) were identified by a *P* value of less than 0.01 and a fold change of at least 2. Compared to the control group, the model group displayed 1988 genes with upregulated expression and 1188 genes with downregulated expression. In the MLCD group, compared to the model group, there were 578 upregulated and 237 downregulated DEGs. KEGG pathway analysis demonstrated the enrichment of DEGs across cell cycle regulation, cancer-related pathways, and the Wnt/β-catenin pathway. Considering the RNA-seq results, we hypothesized that the ability of MLCD to inhibit CRC progression might be linked to Wnt/β-catenin inhibition and cell cycle regulation (Fig. 4a–d).

### Effect of MLCD on the Wnt/β-catenin pathway in intestinal tumor tissues

**Fig. 5:**
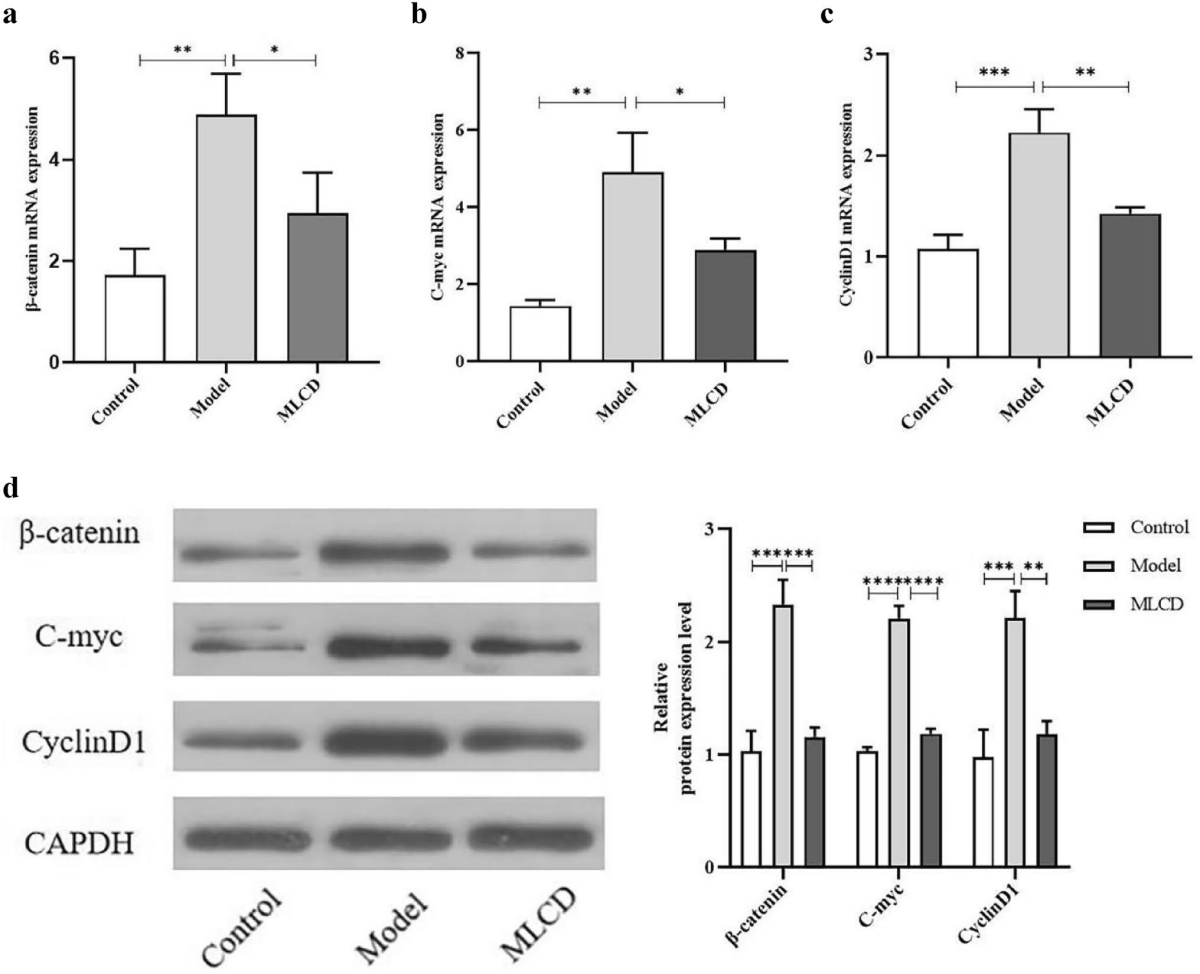
MLCD inhibits CRC progression by regulating Wnt/β-catenin signaling. **a** β-catenin mRNA expression in colorectal tissues. **b** Expression of C-myc mRNA in colorectal tissues. **c** Expression of CyclinD1 mRNA in colorectal tissues. **d** WB analysis of β-catenin, C-myc, and CyclinD1 expression (n = 3). (*P < 0.05, **P < 0.01, ***P < 0.001, and ****P < 0.0001, the difference is statistically significant)

The RNA-seq results suggested that the mechanism by which MLCD inhibits CRC progression may involve the Wnt/β-catenin pathway. To determine whether MLCD inhibits CRC by modulating the Wnt/β-catenin pathway, we examined the protein and mRNA expression of β-catenin, CyclinD1, and C-myc in the intestinal tumor tissues of mice. The expression of these genes was determined using WB and qRT‒PCR. Figure 5a–d shows that the expression levels of β-catenin, CyclinD1, and C-myc were significantly greater in the model group than in the control group (*P* < 0.01). However, the expression of these genes decreased after MLCD treatment.

### Effect of MLCD on the EMT in intestinal tumor tissues

**Fig. 6:**
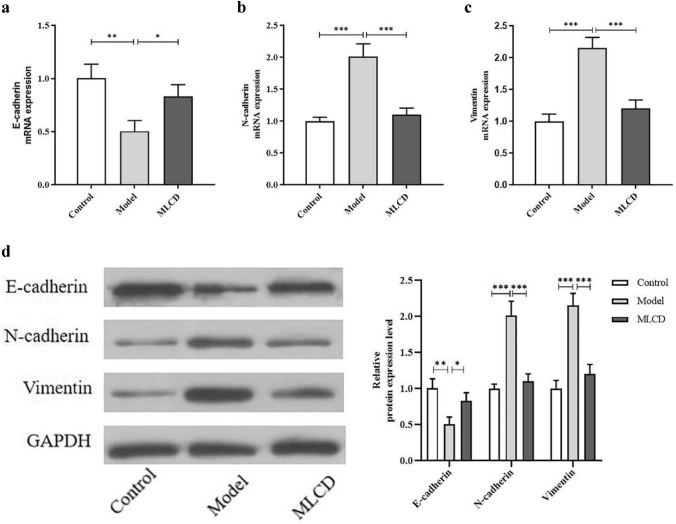
MLCD inhibits CRC progression by regulating the EMT. **a** E-cadherin mRNA expression levels. **b** N-cadherin mRNA expression levels. **c** vimentin mRNA expression level. **d** WB for E-cadherin, N-cadherin, and vimentin (n = 3). (*P < 0.05, **P < 0.01, and ***P < 0.001, the difference is statistically significant)

The nuclear localization of β-catenin and its binding to TCF/LEF are indicative of Wnt/β-catenin pathway activation and serve as mechanisms for promoting the EMT (Gonzalez and Medici 2014). The EMT is a significant hallmark of CRC invasion and metastasis. To validate the impact of MLCD on AOM/DSS-induced EMT in the intestinal tumor tissues of C57BL/6 mice, we assessed the protein and mRNA expression levels of three primary components involved in the EMT process—vimentin, N-cadherin, and E-cadherin—using WB and qRT‒PCR. As depicted in Fig. 6a–d, the model group exhibited elevated levels of vimentin and N-cadherin expression, along with reduced E-cadherin expression, compared to those in the control group. However, MLCD treatment increased E-cadherin expression and decreased N-cadherin and vimentin expression.

### Effect of MLCD on the cell cycle in intestinal tumor tissues

**Fig. 7:**
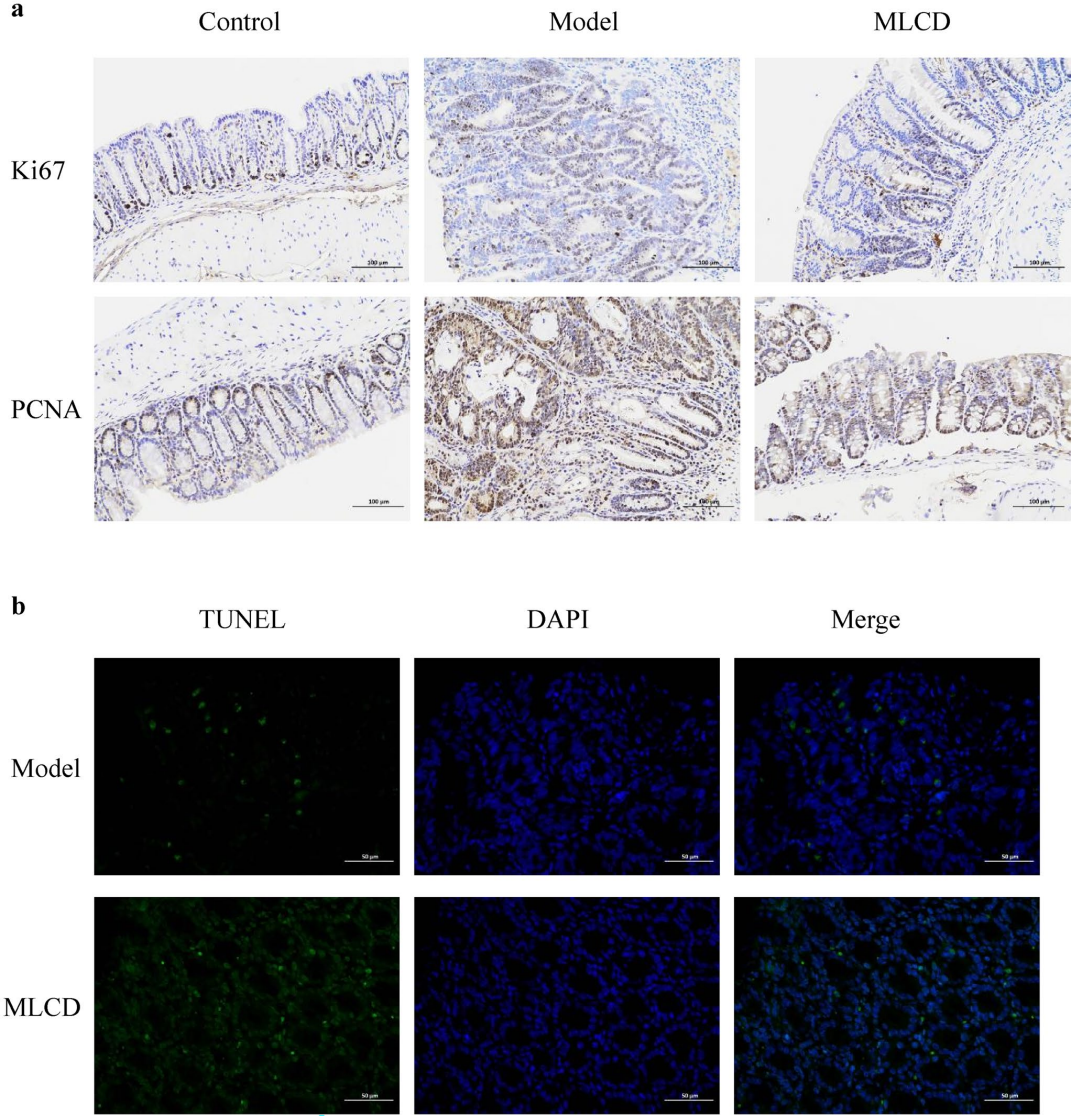
**a** Representative images of Ki67 and PCNA staining in intestinal tissues from the three groups. The scale bar represents 100 µm. **b** TUNEL's images are not left-aligned

Ki67 staining revealed a noticeable presence of Ki67-positive cells (brownish-yellow) in the intestinal tumor tissues of the model group, with a significant concentration in both the muscular and mucosal layers, particularly at the tumor lesion site. There was a reduction in the number of Ki67-positive cells in the MLCD treatment group compared to the model group (Fig. 7a). Furthermore, the model group exhibited a substantial increase in the number of PCNA-positive cells, as characterized by a decrease in the number of deeply stained brown tissue sections; this increase was mitigated by MLCD treatment (Fig. 7a). TUNEL staining revealed that the tumor tissues of the MLCD-treated group exhibited greater levels of apoptosis than did those of the model group (*P* < 0.05) (Fig. 7b).

### MLCD regulates the level of inflammatory cytokines in mice intestinal tissue

**Fig. 8:**
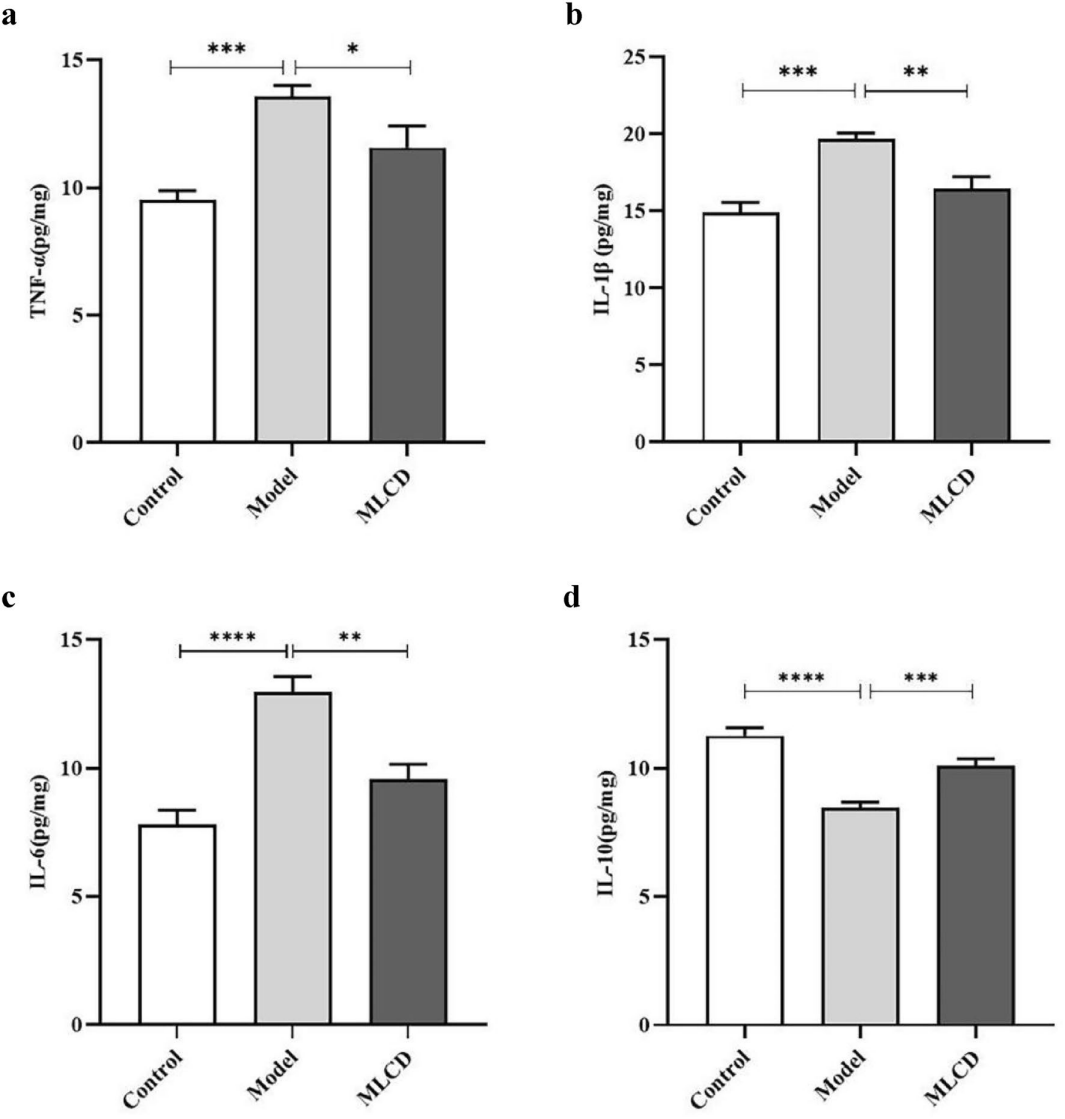
Expression of TNF-α, IL-1β, IL-6, and IL-10 in intestinal tissues. (*P* < 0.05, **P < 0.01, ***P < 0.001, and ****P < 0.0001, the difference is statistically significant)

DSS is a frequently employed drug that is used to establish animal models of ulcerative colitis because it triggers an inflammatory response in the intestinal epithelium, a process that is central to carcinogenesis (Choi et al. 2019). Compared with those in the control group, the model group exhibited significantly elevated levels of proinflammatory factors such as TNF-α, IL-1β, and IL-6 and notably decreased levels of the anti-inflammatory factor IL-10. However, MLCD treatment led to a reduction in TNF-α, IL-1β, and IL-6 levels while increasing IL-10 levels (Fig. 8a–d).

## Discussion

The primary finding of this study was that MLCD inhibited the occurrence and progression of CRC. Pharmacodynamic results indicated that MLCD reduced the number of intestinal tumors, mitigated the extent of pathological damage, and alleviated symptoms such as slower body weight gain and shortening of the intestine in mice. Metagenomic sequencing revealed the ability of MLCD to rectify structural disorders in the intestinal flora. GO and KEGG functional prediction revealed that the unigenes were associated with transcription regulation, DNA templating, signal transduction, transcription, and cell mobility. The RNA-seq findings highlighted a significant enrichment of DEGs in the Wnt/β-catenin pathway and the cell cycle. These molecular biology findings suggest that the mechanism underlying MLCD inhibition of CRC might involve regulation of the Wnt/β-catenin pathway, suppression of abnormal intestinal epithelial cell proliferation, the promotion of apoptosis, and regulation of the EMT.

The Lichong decoction, documented in Zhang Xichun’s Yi Xue Zhong Zhong Can Xi Lu, contains sheng huang qi, dang shen, bai zhu, sheng shan yao, zhi mu, tian hua fen, san leng, e zhu, and ji nei jin. These ingredients aim to strengthen genuine qi and promote blood circulation. The decoction primarily helps address hysteromyomas and has become a popular clinical treatment option for this condition. Contemporary research suggests that the mechanism by which the decoction inhibits uterine fibroids includes hindering abnormal cell proliferation (Li et al. 2012), promoting apoptosis (D. Li et al. 2013) and inhibiting vascular endothelial growth factor expression (Wenna et al. 2020). CRC is a malignant tumor that occurs in the intestines, and its pathogenesis in TCM is similar to that of hysteromyoma, which belongs to the same category of deficiency of positive qi and internal stasis of blood. In addition, we found that dampness-heat is an important causative factor of CRC, so we removed the yin-nourishing and fluid-boosting tian hua fen and zhi mu and added huang lian and huang bai, as well as bai hua she she cao. Huang lian and huang bai have the efficacy of clearing heat and removing toxins, and bai hua sheshe cao can detoxify the body and fight cancer. MLCD is a classic compound formula based on the Lichong decoction, combined with the theoretical basis of CRC pathogenesis and clinical experience.

The intestinal flora maintains direct contact and close interactions with host intestinal epithelial cells. Disturbances in the structure and abundance of these flora have been linked to the development of CRC. Notably, gavage of feces from CRC patients has been shown to activate inflammatory and oncogenic pathways, thereby promoting intestinal carcinogenesis in mice (Wong et al. 2017). In this investigation, we observed a decrease in Muribaculaceae expression in the model group, which subsequently increased following MLCD intervention. Previous research has established a negative correlation between Muribaculaceae abundance and the level of inflammatory cytokines, suggesting that Muribaculaceae is an anti-inflammatory bacterium (Hao et al. 2021). Conversely, the abundance of Bacteroidaceae increased in the model group but decreased after MLCD treatment. Studies have indicated that Bacteroidaceae can promote the growth of CRC cells (Taddese et al. 2020). Sobhani et al. assessed fecal DNA in 179 patients and found elevated levels of Bacteroides/Prevotella (Sobhani et al. 2011). This result aligns with our findings, which demonstrated an increase in Bacteroides and a decrease in Prevotella in the model group. However, importantly, alterations in flora composition merely suggest potential associations with CRC without elucidating the mechanisms by which these changes promote CRC progression. To gain a deeper understanding of the functional implications of the differentially abundant bacteria, we employed GO and KEGG analyses. GO enrichment analyses revealed that the primary BP correlated with these bacteria included the regulation of transcription, particularly DNA templating. The CC and MF terms were associated with cytosol and DNA-binding transcription factor activity, respectively. KEGG pathway analysis suggested that microbial changes were linked to processes such as signal transduction, transcription regulation, and cell motility. These findings align with previous research indicating that the intestinal flora can promote CRC progression through activation of the Wnt/β-catenin pathway (Bennedsen et al. 2022), transcriptional regulation (Pan et al. 2018), and modulation of the EMT (Wan et al. 2018).

The functional predictions based on the aforementioned metagenomic sequencing results included the regulation of transcription, particularly DNA-templated processes. The onset and progression of CRC are known to be associated with an imbalance in oncogenes and tumor suppressor genes. To further elucidate how MLCD suppresses CRC, we conducted RNA-seq analysis to identify DEGs in the control, model, and MLCD-treated groups. Subsequently, we explored the signaling pathways with enrichment of these genes using KEGG enrichment analysis. The results unequivocally indicated that the Wnt/β-catenin pathway was the predominant pathway that was enriched across all three groups. Nearly all CRC patients exhibit aberrant activity in the Wnt/β-catenin pathway (He and Gan 2023). A hallmark of this aberrant activation is the ectopic expression of β-catenin, which translocates from the cell membrane to the nucleus and binds to TCF/LEF within the nucleus (Azzolin et al. 2014). Within this pathway, C-myc and CyclinD1 are major downstream genes. CyclinD1, a crucial regulator of the cellular G1 phase, is highly expressed in more than half of CRC patients (Wei et al. 2019). Moreover, C-myc is a proto-oncogene that promotes the abnormal proliferation of intestinal epithelial cells. Our study revealed that MLCD inhibited the mRNA and protein expression of β-catenin, C-myc, and CyclinD1. These findings suggest that the mechanism by which MLCD inhibits CRC progression is associated with suppression of the Wnt/β-catenin pathway.

Aberrations in the Wnt/β-catenin pathway not only drive the uncontrolled proliferation of intestinal epithelial cells, leading to the formation of intestinal tumors, but also activate the EMT, which further accelerates CRC progression (Vincan and Barker 2008). Changes in the expression of cadherins serve as markers for monitoring the EMT, with downregulation of E-cadherin expression and upregulation of N-cadherin expression being indicative of the EMT (Liu et al. 2016). E-cadherin and N-cadherin are typical markers of epithelial and mesenchymal cells, respectively, and affect adhesion between epithelial cells (Zhang et al. 2021). Similarly, vimentin serves as a marker protein for mesenchymal stromal cells and promotes tumor infiltration and cell migration (Xu et al. 2017). Ma et al. conducted research exploring the mechanism of action of Huangqin-Tang in inhibiting the progression of colitis-associated cancers through proteomics and molecular biology; the results suggested that the mechanism of action may be related to Wnt inactivation and inhibition of the EMT (Ma et al. 2022). In another study, Liu et al. demonstrated that *Lactobacillus fermentum* ZS09 inhibited CRC progression by promoting β-catenin degradation, blocking the Wnt/β-catenin pathway, and subsequently regulating the EMT (Liu et al. 2021). Similar to the above two studies, we employed MLCD to inhibit CRC progression. RNA-seq and KEGG enrichment analysis revealed that the mechanism of action of MLCD involved the Wnt/β-catenin pathway and the cell cycle. Recognizing the pivotal role played by Wnt/β-catenin in regulating the EMT during CRC progression, we conducted WB and qRT‒PCR analyses to assess the levels of E-cadherin, N-cadherin, and vimentin. The results indicated that the MLCD-treated group exhibited higher levels of E-cadherin and lower levels of N-cadherin and vimentin than did the model group. This finding suggested that the mechanism by which MLCD inhibits CRC progression is associated with inhibition of the EMT.

In this study, RNA-seq and KEGG enrichment analyses revealed that the DEGs among the three groups were closely linked to regulation of the cell cycle. The abnormal proliferation and apoptosis of intestinal epithelial cells are key factors in both the initiation and progression of CRC and contribute to malignancy (Brennan and Garrett 2016). Ki67, a marker found in all cell cycle phases except the resting phase, serves as a valuable biomarker for assessing the proliferative activity of cells (Aung et al. 2021; Li et al. 2016). PCNA is widely regarded as an effective marker for detecting cancer cell proliferation (Zhou et al. 2018). TUNEL staining, on the other hand, quantifies apoptotic cells by detecting the color of fragmented DNA. A comparison between the MLCD and model groups revealed a reduced number of Ki67 and PCNA immunohistochemistry-positive particles with lighter staining, while TUNEL staining indicated a significant increase in apoptotic cells in the MLCD group. These findings suggest that the mechanism by which MLCD regulates the cell cycle to inhibit CRC may be associated with inhibiting the abnormal proliferation of intestinal epithelial cells and promoting apoptosis.

IL-10 is known for its anti-inflammatory effects, and a deficiency in IL-10 can lead to spontaneous colitis in mice. Conversely, upregulation of IL-10 expression has been shown to reduce the risk of colitis-associated cancer (Zhang et al. 2016). ELISA data from our study indicated elevated levels of the anti-inflammatory factor IL-10 and reduced levels of the pro-inflammatory factors TNF-α, IL-1β, and IL-6 in the intestinal tissues of MLCD-treated mice. These results suggest that MLCD may inhibit CRC progression by modulating the levels of inflammatory factors.

## Conclusion

Collectively, our results suggest that MLCD might decrease the number of tumors and slow the aggressive progression of CRC. This effect could be connected to the regulation of bacterial flora, deactivation of the Wnt/β-catenin pathway, inhibition of the EMT, and a reduction in the abnormal growth of intestinal epithelial cells while promoting apoptosis. Thus, MLCD could serve as a potential component of TCM prescriptions for CRC treatment.

## Data Availability

The datasets generated during and analysed during the current study are available from the corresponding author on reasonable request.

## References

[CR1] Aung TN, Acs B, Warrell J, Bai Y, Gaule P, Martinez-Morilla S, Vathiotis I (2021). A new tool for technical standardization of the Ki67 immunohistochemical assay. Mod Pathol.

[CR2] Azzolin L, Panciera T, Soligo S, Enzo E, Bicciato S, Dupont S, Bresolin S (2014). YAP/TAZ incorporation in the β-catenin destruction complex orchestrates the Wnt response. Cell.

[CR3] Bennedsen ALB, Furbo S, Bjarnsholt T, Raskov H, Gögenur I, Kvich L (2022). The gut microbiota can orchestrate the signaling pathways in colorectal cancer. APMIS.

[CR4] Brabletz T, Raghu Kalluri M, Nieto A, Weinberg RA (2018). EMT in cancer. Nat Rev Cancer.

[CR5] Brennan CA, Garrett WS (2016). Gut microbiota, inflammation, and colorectal cancer. Annu Rev Microbiol.

[CR6] Choi C-HR, Ibrahim AB, Nik-Sheng D, Gui-Han L, Alan A, Janindra W, Morgan M (2019). Cumulative burden of inflammation predicts colorectal neoplasia risk in ulcerative colitis: a large single-centre study. Gut.

[CR7] Fong W, Li Q, Jun Yu (2020). Gut microbiota modulation: a novel strategy for prevention and treatment of colorectal cancer. Oncogene.

[CR8] Garrett WS (2019). The gut microbiota and colon cancer. Science.

[CR9] Gonzalez DM, Damian M (2014). Signaling mechanisms of the epithelial-mesenchymal transition. Science Signaling.

[CR10] Grivennikov SI, Wang K, Daniel Mucida C, Stewart A, Schnabl B, Jauch D, Taniguchi K (2012). Adenoma-linked barrier defects and microbial products drive IL-23/IL-17-mediated tumour growth. Nature.

[CR11] Hao H, Zhang X, Tong L, Liu Q, Liang Xi, Yushan Bu, Gong P (2021). Effect of extracellular vesicles derived from lactobacillus plantarum Q7 on gut microbiota and ulcerative colitis in mice. Front Immunol.

[CR12] He K, Gan W-J (2023). Wnt/β-catenin signaling pathway in the development and progression of colorectal cancer. Cancer Management and Research.

[CR13] Jin S, Xianguo Wu (2019). Aspirin inhibits colon cancer cell line migration through regulating epithelial-mesenchymal transition via wnt signaling. Oncol Lett.

[CR14] Krishnamurthy N, Kurzrock R (2018). Targeting the Wnt/Beta-catenin pathway in cancer: update on effectors and inhibitors. Cancer Treat Rev.

[CR15] Li D, Zhang Y, Han H, Geng J, Xie X, Zheng J, Wang Y, Zou X (2012). Effect of lichong decoction on expression of IGF-I and proliferating cell nuclear antigen mRNA in rat model of uterine leiomyoma. J Tradit Chin Med.

[CR16] Li D, Xin Xu, Qian R, Geng J, Zhang Y, Xie X, Wang Y, Zou X (2013). Effect of lichong decoction on expression of Bcl-2 and Bcl-2-associated X protein mRNAs in hysteromyoma model rat. J Tradit Chin Med.

[CR17] Li P, Xiao Z-T, Braciak TA, Qing-Jan Ou, Chen G, Oduncu FS (2016). Association between Ki67 index and clinicopathological features in colorectal cancer. Oncol Res Treat.

[CR18] Liu F, Li-Na Gu, Shan B-E, Geng C-Z, Sang M-X (2016). Biomarkers for EMT and MET in breast cancer: an update. Oncol Lett.

[CR19] Liu C-C, Cai D-L, Sun F, Wu Z-H, Yue B, Zhao S-L, Wu X-S (2017). FERMT1 mediates epithelial-mesenchymal transition to promote colon cancer metastasis via modulation of β-catenin transcriptional activity. Oncogene.

[CR20] Liu J, Chen X, Zhou X, Yi R, Yang Z, Zhao X (2021). Lactobacillus fermentum ZS09 mediates epithelial-mesenchymal transition (EMT) by regulating the transcriptional activity of the Wnt/β-catenin signalling pathway to inhibit colon cancer activity. J Inflamm Res.

[CR21] Ma X, Dunfang W, Xue F, Yaqing L, Jia L, Weipeng Y (2022). Huangqin tang interference with colitis associated colorectal cancer through regulation of epithelial mesenchymal transition and cell cycle. Front Pharmacol.

[CR22] Pan W-H, Sommer F, Falk-Paulsen M, Ulas T, Best P, Fazio A, Kachroo P (2018). Exposure to the gut microbiota drives distinct methylome and transcriptome changes in intestinal epithelial cells during postnatal development. Genome Med.

[CR23] Sebio A, Kahn M, Lenz H-J (2014). The potential of targeting Wnt/β-catenin in colon cancer. Expert Opin Ther Targets.

[CR24] Sobhani I, Tap J, Roudot-Thoraval F, Roperch JP, Letulle S, Langella P, Corthier G, Nhieu JTV, Furet JP (2011). Microbial dysbiosis in colorectal cancer (CRC) Patients” ed. Sylviane Pied. Plos One.

[CR25] Sung, H, Jacques F, Rebecca LS, Mathieu L, Isabelle S, Ahmedin Jemal, Freddie B (2021) Global cancer statistics 2020: GLOBOCAN estimates of incidence and mortality worldwide for 36 cancers in 185 countries. CA: Cancer J Clin 71(3): 209–49. 10.3322/caac.21660 10.3322/caac.21660 33538338

[CR26] Taddese R, Garza DR, Ruiter LN, De Jonge MI, Belzer C, Aalvink S, Nagtegaal ID, Dutilh BE, Boleij A (2020). Growth rate alterations of human colorectal cancer cells by 157 gut bacteria. Gut Microbes.

[CR27] Vincan E, Barker N (2008). The upstream components of the wnt signalling pathway in the dynamic EMT and MET associated with colorectal cancer progression. Clin Exp Metas.

[CR28] Wan G, Xie M, Hongjie Yu, Chen H (2018). Intestinal dysbacteriosis activates tumor-associated macrophages to promote epithelial-mesenchymal transition of colorectal cancer. Innate Immun.

[CR29] Wei Y, Huang C, Haoyu Wu, Huang J (2019). Estrogen receptor beta (ERβ) mediated-CyclinD1 degradation via autophagy plays an anti-proliferation role in colon cells. Int J Biol Sci.

[CR30] Wenna W, Zhang W, Li D, Qian R, Zhu L, Liu Y, Chen C (2020). Lichong decoction inhibits micro-angiogenesis by reducing the ex-pressions of hypoxia inducible factor-1α and vascular endothelial growth factor in hysteromyoma Mouse Model. 40(6). 10.19852/j.cnki.jtcm.2020.06.005 33258344

[CR31] Wong SH, Zhao L, Zhang X, Nakatsu G, Han J, Weiqi Xu, Xiao X (2017). Gavage of fecal samples from patients with colorectal cancer promotes intestinal carcinogenesis in germ-free and conventional mice. Gastroenterology.

[CR32] Xu J, Liu D, Niu H, Zhu G, Yangwei Xu, Ye D, Li J, Zhang Q (2017). Resveratrol reverses doxorubicin resistance by inhibiting epithelial-mesenchymal transition (EMT) through modulating PTEN/Akt signaling pathway in gastric cancer. J Exp Clin Cancer Res.

[CR33] Yeung KT, Yang J (2017). Epithelial–mesenchymal transition in tumor metastasis. Mol Oncol.

[CR34] Zhang M, Viennois E, Prasad M, Zhang Y, Wang L, Zhang Z, Han MK (2016). Edible ginger-derived nanoparticles: a novel therapeutic approach for the prevention and treatment of inflammatory bowel disease and colitis-associated cancer. Biomaterials.

[CR35] Zhang N, Ng AS, Cai S, Li Q, Yang Li, Kerr D (2021). Novel therapeutic strategies: targeting epithelial-mesenchymal transition in colorectal cancer. Lancet Oncol.

[CR36] Zhou He, Huang T, Xiong Y, Peng L, Wang R, Zhang GJ (2018). The prognostic value of proliferating cell nuclear antigen expression in colorectal cancer: a meta-analysis. Medicine.

